# The rise of halide perovskite semiconductors

**DOI:** 10.1038/s41377-022-01010-4

**Published:** 2023-01-15

**Authors:** Chenlu He, Xiaogang Liu

**Affiliations:** Department of Chemistry, National University of Singapore, Singapore, 117549 Singapore

**Keywords:** Optical materials and structures, Optical techniques

## Abstract

Advances in metal-halide perovskite semiconductors have significantly influenced light-current conversion technologies. The excellent structural and compositional tunability of perovskites, coupled with their exceptional quantum yields for photoluminescence, make them a promising candidate for optoelectronic devices.

Metal-halide perovskites, a unique class of outstanding photosensitive semiconductors, are widely employed in solar cells, light-emitting diodes (LEDs), photodetectors, lasers, and X-ray scintillators due to their excellent photophysical properties and ease of processin in solution^1–6^. Tremendous efforts have led to dramatic developments in perovskite-based devices over the past decade, but their adoption into future optoelectronics remains a challenge^7^.

Now, Dong et al. review the historical milestones of research on metal-halide perovskites and their revolutionary impact on optoelectronics^8^. The unique optoelectronic properties of perovskites include high optical absorption, high carrier mobility, long diffusion lengths, and unique ambipolar charge transport properties. Perovskites possess these advantages because of their distinct crystal structure and chemical composition, which can be modified by their phase, dimension, composition, and geometry. Consequently, perovskites can be used for a wide range of optoelectronic needs, primarily serving as light emitters or transducers. This gives them unprecedented flexibility to tune their optoelectronic properties independently and synergistically^9^.

Light harvesting devices, such as photodetectors, can convert incident photons into free charge carriers in metal halide perovskites. In this review, it is briefly explained that photodetectors can be classified into vertical and lateral structures, which are different from the typical sandwich architecture of solar cells^10^. As a light-emitting layer, the external quantum efficiency of LEDs has now exceeded 20%^11,12^. Moreover, a stable continuous-wave pumped perovskite laser with amplified spontaneous emission has been demonstrated at room-temperature, marking a significant advancement in engineering electrically pumped lasers and integrated optoelectronics^13^.

The behavior of photogenerated carriers is primarily governed by light-matter interactions, such as absorption, emission, modulation and transmission, at the heart of an optoelectronic device. In general, optoelectronic devices correspond to various photophysical processes that have their own suitable working conditions, including excitation density and charge carrier density. This review summarizes the nature of photogenerated excitons/carriers in perovskites and explains the mechanism of the photophysical process in various optoelectronic devices (Fig. 1).

**Fig. 1:**
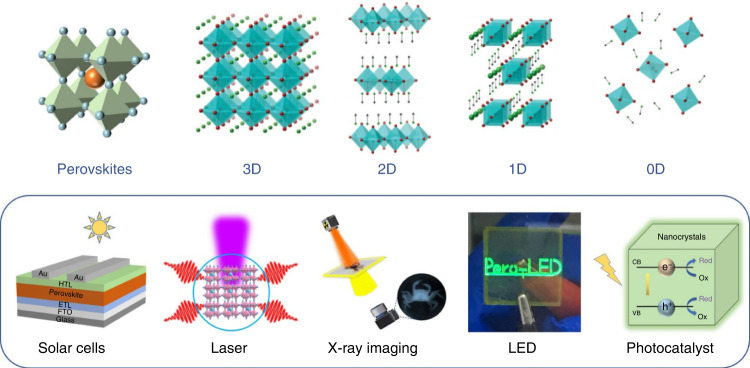
Halide Perovskite Semiconductors Schematic representation of perovskite frameworks with different dimensionalities (3D, 2D, 1D, and 0D) and various applications enabled by perovskite-based optoelectronic components

There have been many reports on perovskite-based optoelectronics in various fields due to their unique optoelectronic properties. Dong et al. divide these applications into four sections, including functional integration, information display, electronic communication, health and medical systems. They highlight two current research directions on perovskites. One is scintillator materials that allow facile visualization for X-ray imaging^14^. Another direction is the development of optoelectronic devices based on chiral perovskites^15^. Chiral hybrid organic–inorganic perovskites have shown promise as materials for chiroptoelectronics, including photodetectors for circularly polarized light, 3D displays, and spintronics, as well as applications in quantum communications and quantum computing^16^. Furthermore, they provide an outlook on future directions of this field by discussing long-term instability and toxicity of perovskites. This review is a very timely and covers a very promising area of research, giving a wonderful overview of the remarkable optoelectronic properties and applications of metal-halide perovskites.

Although great progress has been made in perovskite materials, we believe there is still a long way from commercial applications. For example, in integrated optoelectronics, structured perovskite optoelectronics with versatile functionalities remains a challenge. Therefore, the development of multifunctional micro- or nanostructures for perovskite optoelectronics is an effective approach to integrating structures and properties. Meanwhile, optoelectronic applications based on perovskites have to withstand increasingly complex environmental conditions, such as high temperature, humidity or ultraviolet irradiation. This poses a major challenge to the stability of perovskite devices. For example, it is necessary to develop perovskite materials for flexible X-ray scintillator screens^16,17^. Besides providing high-quality imaging on non-flat objects, these screens can also handle non-uniform X-rays under special circumstances, alleviating the problem of vignetting on large-area objects caused by uneven dose distribution. Despite these challenges, we believe perovskite semiconductors have a bright future thanks to their excellent optoelectronic properties.
